# The Impact of Pine Wood Nematode Infection on the Host Fungal Community

**DOI:** 10.3390/microorganisms9050896

**Published:** 2021-04-22

**Authors:** Yi Liu, Zhao-Lei Qu, Bing Liu, Yang Ma, Jie Xu, Wen-Xiao Shen, Hui Sun

**Affiliations:** 1 Collaborative Innovation Center of Sustainable Forestry in Southern China, College of Forestry, Nanjing Forestry University, Nanjing 210037, China; liuyi1419414481@163.com (Y.L.); qzl941211@njfu.edu.cn (Z.-L.Q.); 202404324@163.com (B.L.); mayang0524@outlook.com (Y.M.); xujie_njfu@163.com (J.X.); 2 School of Foreign Language, Nanjing University of Finance and Economics, Nanjing 210046, China; kenyshen@163.com

**Keywords:** *Bursaphelenchus xylophilus*, pine wilt disease, fungal-community structure, functional structure, microbe-host interaction

## Abstract

Pine wilt disease (PWD), caused by pinewood nematode (PWN) *Bursaphelenchus xylophilus*, is globally one of the most destructive diseases of pine forests, especially in China. However, little is known about the effect of PWD on the host microbiome. In this study, the fungal community and functional structures in the needles, roots, and soil of and around *Pinus thunbergii* naturally infected by PWN were investigated by using high-throughput sequencing coupled with the functional prediction (FUNGuild). The results showed that fungal richness, diversity, and evenness in the needles of diseased trees were significantly lower than those of healthy ones (*p* < 0.05), whereas no differences were found in the roots and soil. Principal coordinate analysis (PCoA) showed that the fungal community and functional structures significantly differed only in the needles of diseased and healthy trees, but not in the soil and roots. Functionally, the saprotrophs had a higher abundance in the needles of diseased trees, whereas symbiotrophs abundance was higher in the needles of healthy trees (linear discriminant analysis (LDA) > 2.0, *p* < 0.05). These results indicated that PWN infection primarily affected the fungal community and functional structures in the needles of *P*. *thunbergii*, but not the roots and soil.

## 1. Introduction

Plant-associated microbes refer to total microorganisms that colonize the surface and interior of a plant, including fungi, bacteria, and archaea, and can be divided into phyllosphere, endosphere, and rhizosphere according to the different parts of plants [1,2]. They can interact with the host plant in a beneficial, harmful, or neutral way [3,4,5]. However, studies on forest tree microbiomes and their roles in mutualism and disease lag far behind parallel work on crop and human microbiome projects [6]. The recent and fast development in sequence technology provides the possibility to better understand plant and tree microbiomes. The tree-associated endophytic fungi play important functional roles and impact on forest tree health via producing alkaloid toxins or enhancing plant ecophysiology against some biotic and abiotic stresses [6,7]. In addition, many ectomycorrhizal or ericoid mycorrhizal fungi can form symbiotic relationships and interact with plants [8]. Some fungi can affect plant growth by producing plant hormones to manipulate plant regulatory pathways, or increasing nutrient availability from the environment to promote the resistance or tolerance of the host to biotic and abiotic stresses [9,10,11]. On the contrary, opportunistic pathogens often lead to plant diseases under certain circumstances and have negative effects on host health [12]. For example, *Diplodia sapinea*, a common endophytic fungus, can cause conifer tip blight and form perennial cankers that can lead to sudden branch death [13,14]. Moreover, previous studies have shown that Basidiomycota is the dominant fungal group in bulk soil [15], whereas Ascomycota is the most prevalent group in the needle tissues [16], suggesting the significant difference in microbial distribution between forest soil and plant tissues.

Many factors can affect the plant microbiome, such as the composition and abundance of host plant species and environmental factors [17,18]. Previous studies showed that different plant species harbored distinct plant-associated fungi [19]. Microbial communities in different anatomical parts of the plant also differed due to differences in the structure and chemical composition [4,20], e.g., mycorrhizae are more common in plant roots [21], and Sordariomycetes is the dominant class in the stem of *Eleutherococcus senticosus* (also *Acanthopanax senticosus*) [22]. In addition, the health status of the host plant may influence plant-associated microbial communities and certain specific microbial groups [23]. Plant disease can reduce host health status, which—in turn—affects the plant-associated microbial community. A study of pea root rot disease found that *Epicoccum nigrum* almost exclusively disappeared in diseased roots, and *Exophiala salmonis* decreased in abundance [24]. Fungal richness and diversity, and the abundance of *Lophodermium* in the diseased needles of *Pinus sylvestris* significantly increased when compared to in healthy ones [25]. Previous studies also investigated endophytic fungi in declining trees or a specific part of a plant, such as leaves, twigs, or roots [26,27,28], but studies on disease-induced changes in host-fungal communities are rare. Therefore, it is important to understand the response of a host-associated microbiome to disease attack, which could help to better understand the relationship between host, host-associated microbes, and plant disease.

Soil microbes represent the greatest reservoir of biological diversity [29,30] that can interact with the plant. Soil fungi can degrade soil organic matter and play a key role in nutrient cycling due to their ability to produce a wide range of hydrolytic and oxidative enzymes [20,31,32,33]. Soil saprophytic fungi perform the initial steps in the decomposition of cellulose, lignin, and other complex macromolecules, and then the process is further processed by bacteria [34]. Symbiotic fungi such as ectomycorrhizal (ECM) fungi, can help plants absorb nutrients [35,36] to elevate host resistance against diseases and parasites [37,38]. In return, the plant provides carbon (C) for microbial growth [39,40]. However, many factors could affect soil fungal communities, such as soil properties, plant species, and plant health status [41,42]. Wan (2019) showed that soil fungal diversity and composition differed in plantations with *Bupleurum chinense* and *P. tabulaeformis* [43]. Plant disease can reduce the host health and change or activate root exudates, which shift below-ground microbial communities [40]. However, most studies on disease-induced plant microbes were limited to agricultural crops, and in comparison, the consideration and characterization of the microbial communities in forest trees are still in their infancy [6].

Pine wilt disease (PWD), caused by pinewood nematode (PWN) *Bursaphelenchus xylophilus*, is one of the most destructive diseases of pine forests, which causes significant economic losses in Asian and European countries, such as China, Japan, and Portugal [44,45,46]. The disease mechanism is very complicated as many agents are involved including the nematode, transmission vector, and bacteria associated with nematode and host [44]. Briefly, *B*. *xylophilus* enters the xylem of the host through the wound when the *Monochamus alternatus* (vector) fly and feed on the branches of healthy pine trees in early spring, parasitizes in the resin channel, and gradually spreads throughout the whole plant. *Pinus* spp. affected by PWD are characterized by a decrease of resin flow and browning/reddening needles, as a result of water transportation mechanisms damaged due to *B*. *xylophilus*, which decreases the health of the total tree rapidly even death within few months [47]. Recent studies mainly focused on the pathogenic mechanism of PWD [48,49,50], but information on the response of plant microbial communities to a PWN infection is still limited. Previous studies showed that the fungal species that were dominant in *P. thunbergii* had a compatible relationship with PWN to increase nematode reproduction, while some had an incompatible or neutral relationship [51,52]. PWN infection can alter root-associated fungal communities in *P*. *tabulaeformis,* and alter the rhizosphere and endophytic microbial community of *P*. *massoniana* [53,54]. A recent study showed that PWD only affects bacterial community structure and functional structure in roots and needles, respectively, and did not affect host bacterial diversity [55]. In the current study, we selected *P. thunbergii* Parl. trees naturally infected by PWN to study the host fungal community and functional structures using high-throughput Illumina-Miseq sequencing coupled with FUNGuild (http://www.stbates.org/guilds/app.php) functional analysis [56]. The study aims to elucidate how PWD affects the host fungal community and functional structure, and provide useful information on the interaction between disease-induced forest decline and plant microbiome.

## 2. Materials and Methods

### 2.1. Study Sites and Sample Collection

Samples were collected from the Sun Yat-sen’s Mausoleum Park, which is located in Purple (Zijin) Mountain, Nanjing, China (32°04′ N, 118°50′ E). Detailed information on the sites and sample collection was previously outlined [55]. Briefly, the study area covers approximately 30 km^2^, and is part of a subtropical transition zone with average annual sunshine of 1628.8 h, an average temperature of 19.6 °C ranging from −3.7 °C in winter to 38.8 °C in summer, and average annual precipitation of 1530.1 mm ranging from 1091 to 2371.4 mm. The average relative humidity of the previous year is 76%, ranging from 81% to 73%, the annual change is big in June and July, and the change in April, May, August, and September is small [57]. Forests were originally dominated by conifer trees of *P. thunbergii* and *P*. *massoniana* Lamb, which are currently aged around 70 years. Due to the continuous spread of pine wilt disease, a large number of susceptible pine trees were killed, resulting in the placement of secondary broad-leaved forests, including *L*. *formosana* Hance (sweetgum) and *Q*. *acutissima* Carruth (Oak). Vegetation includes shrubs (*Symplocos paniculata*, *Camellia sinensis*, and *Lindera glauca*) and herbs (*Ophiopogon japonicus*, *C. communis*, and *Reynoutria japonica*) above ground. There were three study plots of 20 × 20 m each and 500 m apart. Three diseased *P. thunbergii* trees with obvious symptoms of PWD and three healthy trees were chosen in each plot. Diseased trees refer to trees infected by *B. xylophilus* but still alive on the last stage of disease development. Diseased trees showed typical PWD symptoms, with most of the needles turning brown in September. Healthy trees refer to those with completely green needles and no signs of PWN infection. The distance between diseased and adjacent healthy trees was less than 15 m. Subsequent confirmation of healthy and diseased trees was carried out in the laboratory by the isolation of nematodes and PCR amplification with DNA extracted from the trees using specific primers of *B. xylophilus* [58].

The needles and roots of each tree, and the soil surrounding the trees were sampled. Fifteen needles from each tree were collected from the middle of the crown after the tree was felled. Needles were collected from three directions (120° as the boundary) and mixed as one sample. Root samples were obtained from the main root below the soil surface, about 25 cm depth, with a sterilized puncher (10 mm in diameter) from three directions (120° as the boundary). Three samples from each tree were mixed as one composite sample. In total, 18 samples from needles and roots (9 diseased samples and 9 healthy ones) were obtained, respectively. Due to high heterogeneity, three soil samples surrounding each tree were collected. The soil was collected from the three directions (with 120° as the boundary) of each tree, representing three soil samples. From each direction, three subsoil samples were collected at a distance of 20, 40, and 60 cm from the tree trunk after litter removal, and then mixed as one composite soil sample. In total, 54 soil samples (3 directions from the tree × 6 trees/plot × 3 plots = 54 soil samples) were collected. Samples were delivered to the lab on ice and kept at −20 °C for subsequent analysis. The soil physical and chemical properties around diseased and healthy trees were published in our previous study by Ma et al. [55] and were cited as supplementary Table S1.

### 2.2. DNA Extraction, PCR Amplification, and Illumina MiSeq Sequencing

Following the manufacturer’s instructions, genomic DNA from the soil was extracted using Soil DNA kits (OMEGA BIO TEK, Norcross, GA, USA), and genomic DNA from the needles and roots was extracted using Plant Genomic DNA Kits (TIANGEN BIOTECH (BEIJING) CO., LTD, Beijing, China). The procedure was previously described in detail [55]. A NanoDrop ND-1000 spectrophotometer (Thermo Fisher Scientific, Wilmington, DE, USA) was used to measure DNA concentrations. Fungal internal transcribed spacer 1 (ITS1) was amplified using primer set ITS1-F (5’-CTTGGTCATTTAGAGGAAGTAA-3’) and ITS2-R (5’- GCTGCGTTCTTCATCGATGC-3’) [59]. PCR was performed in triplicate for each sample in a TransGen AP221-0220 µL reaction system. The reaction included 4 µL 5 × FastPfu Buffer, 2 µL dNTPs (2.5 mM), 0.8 µL Forward Primer (5 µM), 0.8 µL Reverse Primer (5 µM), 0.4 µL FastPfu Polymerase, 0.2 µL Bovine serum albumin (BSA), and 10 ng Template DNA. The PCR reaction parameters were as follows: 95 °C for 3 min, 27 cycles of 95 °C for 30 s, annealing temperature of 55 °C for 30 s, 72 °C for 45 s, and a final extension of 10 min at 72 °C. Negative PCR with sterilized water as template was included to track possible contaminations. The PCR product was detected using 2% agarose gel electrophoresis, and purified with Agencourt AMPure XP beads (Beckman Coulter, Pasadena, CA, USA). Concentration was measured using a NanoDrop ND-1000 spectrophotometer and subjected for sequencing with a paired-end (PE = 300) Illumina MiSeq platform at Majorbio (Shanghai International Medical Zone, China). Raw sequences were deposited at the Sequence Read Archive (SRA) of the National Center for Biotechnology Information (NCBI) under project accession number PRJNA703504.

### 2.3. Bioinformatics and Statistical Analysis

Mothur software was used to process the raw sequence data according to the Standard Operating Procedure (SOP) [60]. Briefly, the adapter and barcode sequences were removed using Cutadapt v.1.15. Sequences were denoised and quality checked for sequencing errors (trim.seqs), PCR errors (pc.seqs), and chimera (chimera.uchime) using mothur commands. Sequences were then pairwise-aligned using the pairwise.seq command, and preclustered with 2 base-pair differences to remove sequences that were likely due to sequencing errors. Sequences were then clustered to operational taxonomic units (OTUs) at 97% similarity [61]. The most abundant sequence in each OTU was selected as the representative sequence for the OTU assignment. High-quality and unique sequences were assigned to a taxonomic group with 80% bootstrap confidence by using the mothur-formatted UNITE taxonomy reference database (UNITE + INSD, version 8.0) [62]. Sequences assigned to the plant chloroplast and nonfungal domain were filtered out.

To correct differences in sample size, a rarified subset of data with the smallest size of the sample across all datasets were analyzed to calculate the diversity index including α diversity (Shannon), species richness (Chao1), evenness (Shannon evenness), and Good’s coverage to ensure comparable comparison across samples [55]. One-way ANOVA tests were used to identify differences in community diversity index (richness, α diversity, and evenness) by IBM SPSS Statistics 20.0 software. Venn diagrams were constructed using subsampled data to show shared and unique OTUs with InteractiVenn (http://www.interactivenn.net (accessed on 18 December 2019)) [63].

FUNGuild (Fungi + Functional + Guilds, http://www.stbates.org/guilds/app.php (accessed on 31 September 2019)), a Python-based tool to taxonomically parse fungal OTUs into ecological guilds, was used to predict the community functional structure. Fungus OTUs were divided into three trophic modes, namely, pathotrophs, symbiotrophs, and saprotrophs [56]. Linear discriminant analysis (LDA) effect size (LEfSe; http://huttenhower.sph.harvard.edu/galaxy (accessed on 9 July 2020)) was used to identify fungal taxonomic and functional groups differentially represented between diseased and healthy treatments [64]. Principal coordinate analysis (PCoA) and canonical correspondence analysis (CCA) were used to visualize differences in fungal community and functional structure, respectively, followed by confirmation with permutational multivariate analysis of variance (PERMANOVA) in PRIMER 7 [65].

## 3. Results

### 3.1. Information on Illumina MiSeq Sequencing Data

A total of 4,520,049 high-quality sequences were generated across all (90) samples after sequence denoising and quality filtering. The average number of sequences per sample was 50,223 ± 1300 (mean ± standard deviation), ranging from 14,692 to 75,144 per sample. The smallest sample size (14,692 sequences) was used to randomly rarify the dataset for calculating the diversity index and community structure. Good’s coverage of sequence was more than 98% for each sample, and sequencing depth effort is shown in Figure S1 as a rarefaction curve.

### 3.2. Fungal Diversity Index between Diseased and Healthy Trees

**Figure 1:**
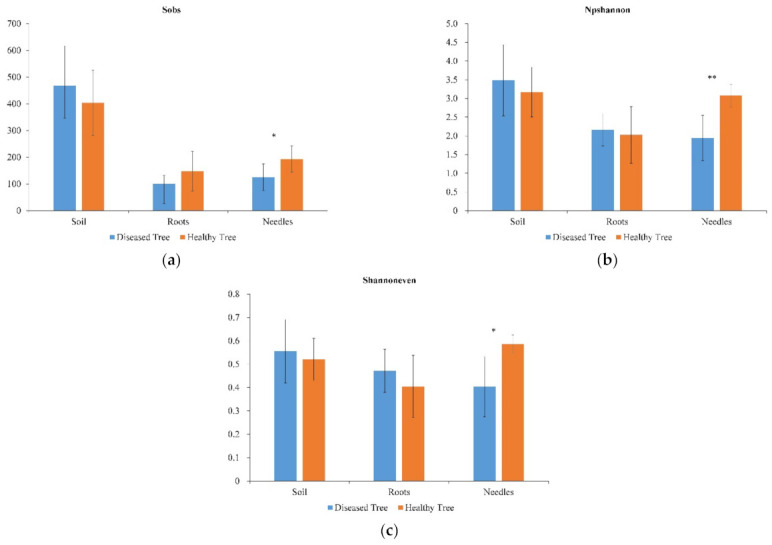
Diversity indices of fungal community in soil, roots, and needles around and of healthy and diseased trees. (**a**) Species richness, (**b**) Shannon α diversity, and (**c**) Shannon evenness. Values show mean with standard deviation (*n* = 9, except *n* = 27 for soil); * *p* < 0.05 and ** *p* < 0.01.

Fungal species richness, α diversity, and evenness in the needles of diseased trees were significantly lower than those of healthy trees (*p* < 0.05; Figure 1). However, no significant differences in the fungal diversity index were found in either the soil or roots of healthy and diseased trees (Figure 1).

### 3.3. Fungal-Community Structure on OTU Level

**Figure 2:**
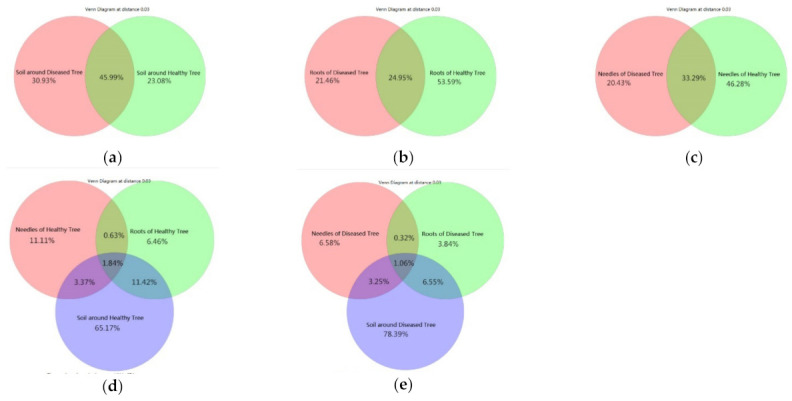
Venn diagram showing unique and shared operational taxonomic units (OTUs) between healthy and diseased samples in (**a**) soil, (**b**) roots, and (**c**) needles, and among soil, roots, and needles in (**d**) healthy and (**e**) diseased trees.

In total, 6667 OTUs were obtained across all samples. The number of shared and unique OTUs differed between diseased and healthy trees in the soil, roots, and needles (Figure 2). The soil had the highest number of the shared OTUs (46.0%), followed by needles (33.3%) and roots (24. 5%) (Figure 2a–c). The number of unique OTUs in heathy roots or needles was more than double that of the diseased trees (Figure 2b,c). However, the number of unique OTUs in the soil around healthy trees was lower than that around diseased trees (Figure 2a). Only 1.1% and 1.8% of OTUs were shared among the soil, roots, and needles in diseased and healthy trees, respectively (Figure 2d,e). The soil had the highest number of unique OTUs in both healthy and diseased samples, followed by needles and roots.

**Figure 3:**
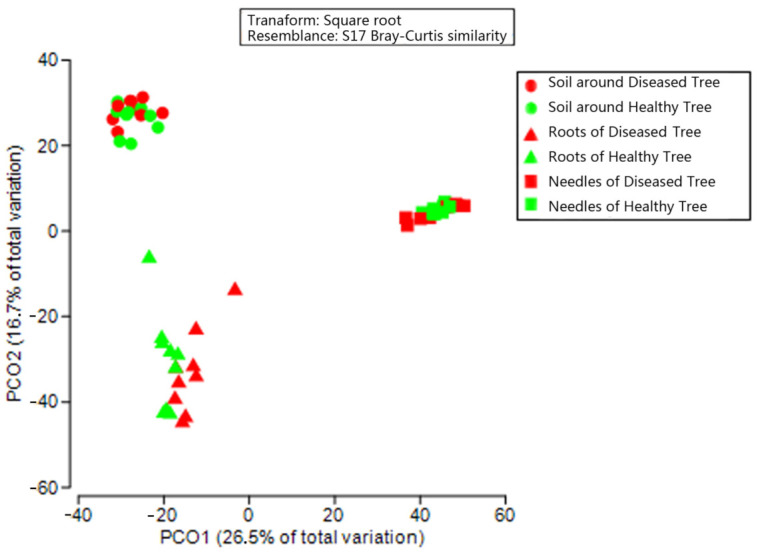
Principal-coordinate analysis (PCoA) showing fungal community structure in soil, roots, and needles around and of healthy and diseased trees.

PCoA based on OTU data detected 43.2% of total variation among fungal communities, with the first and second axes explaining 26.5% and 16.7% of variation, respectively (Figure 3). The fungal community structure between healthy and diseased trees differed only in needles. Subsequent PERMANOVA confirmed the structural difference (*p* < 0.05; Table S2). The top 10 OTUs (>0.1%) in the needles showing significant differences between healthy and diseased trees, and contributing to the structural difference are shown in Table 1: Trichomeriaceae (OTU00019, 00026, 00040, 00063, and 00070) and *Strelitziana* (OTU00025 and 00028) had higher abundance in the needles of healthy trees, covering 7 OTUs; whereas *Diplodia* (OTU00007), *Phacidium* (OTU00011), and *Hormonema macrosporum* (OUT00030) were more abundant in the needles of diseased trees. No differences in fungal community structure were found in either roots or soil of healthy and diseased trees (Figure 3). The soil, roots, and needles also formed distinct fungal communities (*p* < 0.05 in all possible pairs).

### 3.4. Fungal Community Structure on Taxonomic Level between Diseased and Healthy Trees

**Figure 4:**
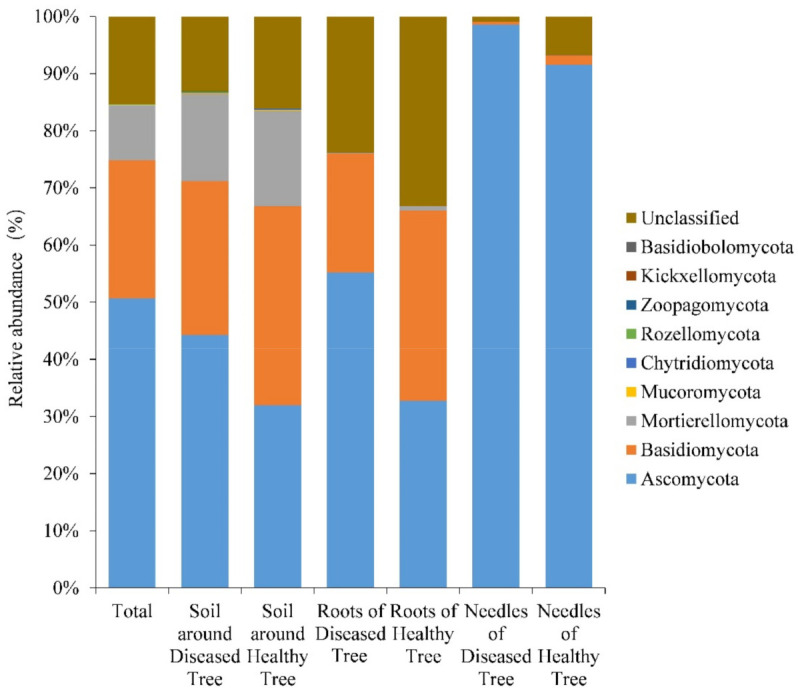
Relative abundance of fungal phyla in soil, roots, and needles around and of healthy and diseased trees.

All sequences were classified to the fungal domain and assigned to 6667 OTUs comprising 11 phyla, 528 genera, and 650 species. On the phylum level, the most predominant was Ascomycota with 50.7% of sequences and 54.6% of the OTUs, followed by Basidiomycota (24.2% of sequences and 13.8% of OTUs), and Mortierellomycota (9.5% of sequences and 1.8% of OTUs) (Figure 4). Minor phyla (≤0.1% of sequences) were Mucoromycota, Chytridiomycota, Rozellomycota, Zoopagomycota, Kickxellomycota, and Basidiobolomycota (Figure 4).

On the genus level, *Mortierella* was the most abundant (9.3% of sequences and 1.6% of OTUs), followed by *Delicatula* (3.9% of sequences and 0.5% of OTUs), *Trichoderma* (3.1% of sequences and 3.7% of OTUs), *Diplodia* (1.9% of sequences and 0.1% of OTUs), *Solicoccozyma* (1.9% of sequences and 0.3% of OTUs), and *Cenangium* (1.9% of sequences and 0.2% of OTUs) (Table S3). On the species level, abundant species included *Mortierella humilis* (13.9% of sequences and 0.1% of OTUs), *M. minutissima* (5.5% of sequences and 0.3% of OTUs), *Pestalotiopsis rhododendri* (4.0% of sequences and 0.2% of OTUs), *Solicoccozyma terrea* (3.9% of sequences and 0.1% of OTUs), *Hormonema macrosporum* (2.1% of sequences and 0.1% of OTUs), and *Humicola olivacea* (1.5% of sequences and 0.2% of OTUs) (Table S4).

**Figure 5:**
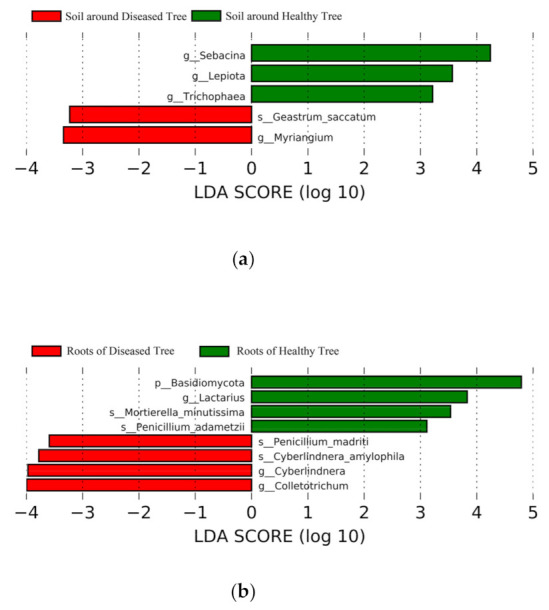

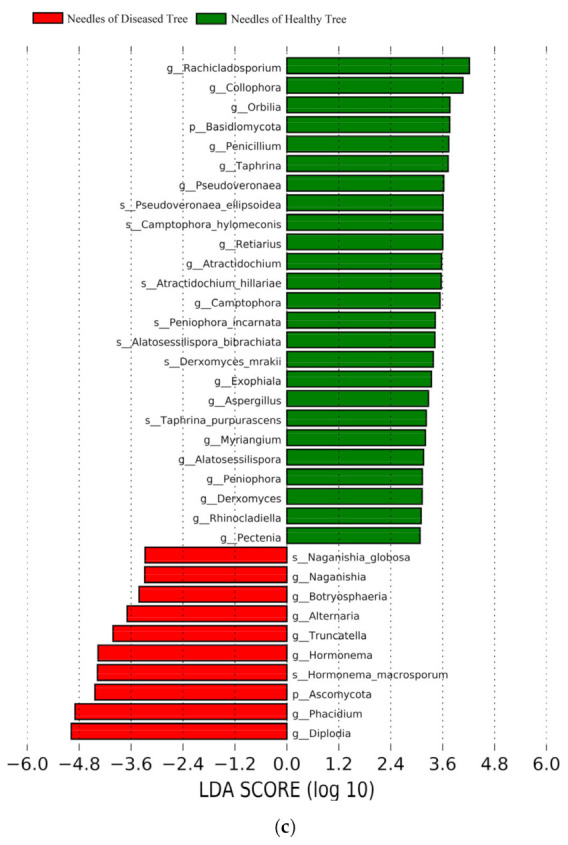
Linear discriminant analysis (LDA) effect size (LEfSe) showing fungal phyla, genera, and species that significantly differed in (**a**) soil, (**b**) roots, and (**c**) needles around and of healthy and diseased trees. Abbreviations: p, phylum; g, genus; s, species.

Some taxonomic groups on different levels differed between diseased and healthy trees in the soil, roots, and needles, respectively (LDA > 3.0, *p* < 0.05). In the soil, genera *Sebacina*, *Lepiota*, and *Trichophaea* were more abundant in healthy trees, whereas the abundance of fungi of the *Myriangium* genus and the *Scytalidium lignicola* species was higher in diseased trees (Figure 5a). In the roots, the Basidiomycota phylum, *Lactarius* genus, and *M. minutissima* and *Penicillium adametzii* species had higher abundance in healthy trees, whereas the *Colletotrichum* and *Cyberlindnera* genera, and the *Cyberlindnera amylophila* and *P. madriti* species were more abundant in diseased trees (Figure 5b). In the needles, the Basidiomycota phylum; 17 genera, including *Rachicladosporium*, *Penicillium*, and *Rhinocladiella*; and 7 species, including *Pseudoveronaea ellipsoidea* had higher abundance in healthy needles (Figure 5c), whereas the Ascomycota phylum; 7 genera, including *Diplodia* and *Hormonema*; 2 species, *H. macrosporum* and *Naganishia globose,* were more abundant in the needles of diseased trees.

### 3.5. Potential Fungal Functional Structure between Diseased and Healthy Trees

**Figure 6:**
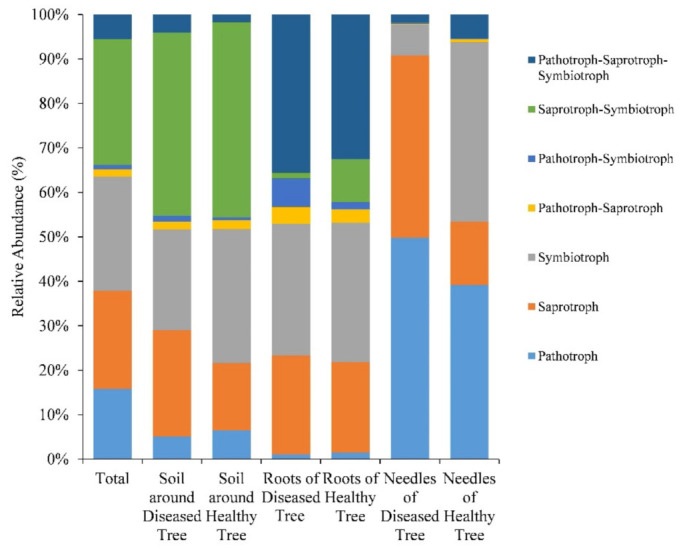
FUNGuild (http://www.states.org/guilds/app.php (accessed on 31/9/2019)) analysis showing predicted trophic mode in soil, roots, and needles around and of healthy and diseased trees.

Of total OTUs, 33.3% (2217 OTUs), covering 48.0% (2,170,790 sequences) of the total sequences could be assigned to trophic modes and functional guilds with highly probable or probable confidence. Symbiotrophs were the most abundant group, accounting for 25.7% of the reads, followed by saprotrophs (22.0%) and pathotrophs (15.9%). Saprotroph–symbiotroph accounted for 28.2% of the reads, and pathotroph–saprotroph–symbiotroph, pathotroph–saprotroph, and pathotroph–symbiotroph accounted for 5.6%, 1.6%, and 1.1%, respectively (Figure 6).

**Figure 7:**
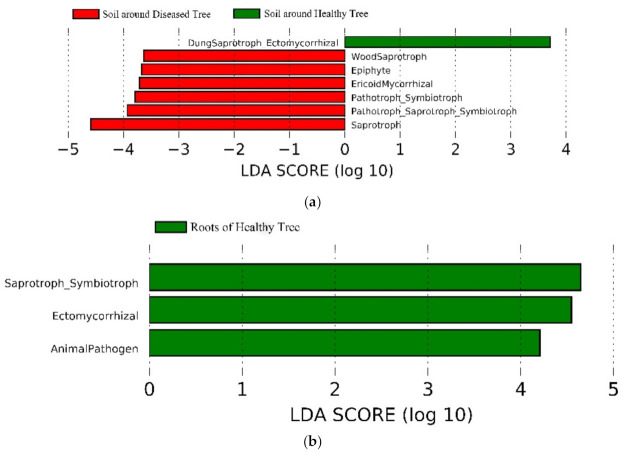

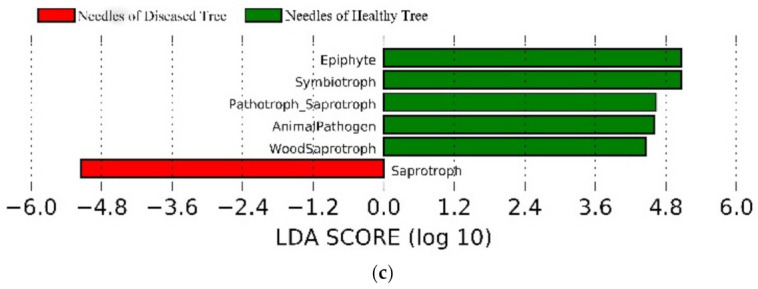
LEfSe (LDA > 2.0, *p* < 0.05) showing predicted functional groups significantly presented in (**a**) soil, (**b**) roots, and (**c**) needles around and of healthy and diseased samples.

LEfSe showed that the abundance of certain functional groups differed between diseased and healthy samples in the soil, roots, and needles, respectively (LDA > 2.0, *p* < 0.05) (Figure 7). The abundance of saprotrophs and ericoid mycorrhizal, epiphytes, and wood saprotrophs was higher in the soil surrounding diseased trees (Figure 7a). In the needles, saprotrophs had a higher abundance in diseased trees, whereas the abundance of symbiotrophs, epiphytes, wood saprotrophs, and animal pathogens was higher in healthy ones (Figure 7c). The abundance of saprotroph–symbiotroph, ectomycorrhizal, and animal pathogens was higher in the roots of healthy trees (Figure 7b).

**Figure 8:**
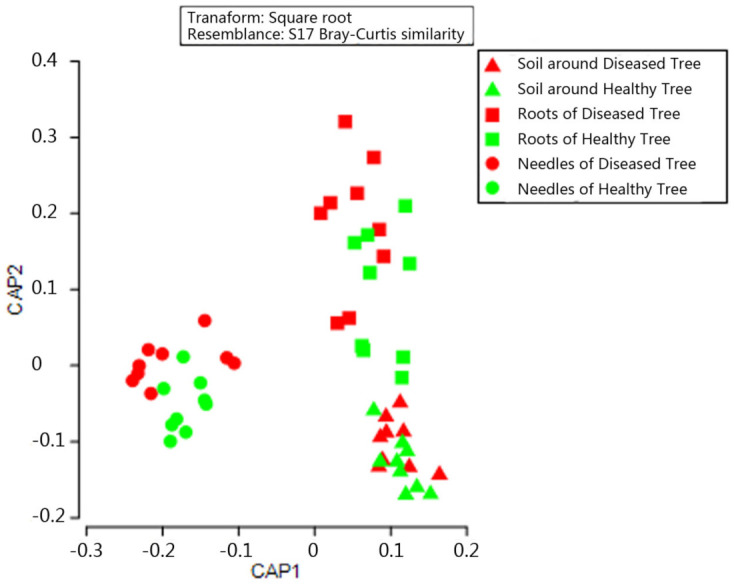
Canonical correspondence analysis (CCA) showing a fungal functional structure in soil, roots, and needles around and of healthy and diseased trees.

Similar to community structure, CCA showed that the fungal functional structures differed only in the needles of healthy and diseased trees (*p* < 0.01), but in neither the soil nor the roots (Figure 8). Subsequent PERMANOVA confirmed the difference in functional structure in the needles (*p* < 0.05; Table S5).

## 4. Discussion

In this study, we investigated the fungal communities and potential functional structures in host trees and the soil after PWN infection in the field. Overall, PWD significantly decreased fungal α diversity in the needles, which is in line with previous observations that the tree disease can lower phyllosphere fungal diversity [66,67]. Compared to diseased trees, phyllosphere microbes in healthy trees may form larger and complex networks containing significantly greater microbial consortia, e.g., some well-known biocontrol agents (BCAs) and more functional genes, which probably provides stabler and more beneficial conditions to plants [67]. Microenvironmental conditions and nutrient supply in the phyllosphere can also affect the colonization of some endophytic fungi, such as *Lophodermium* [68]. In senescent and dead leaves with weak host defense ability, nutrient reserves in the leaves decreased, and some fungi with strong competitive ability may replace other endophytes, which might cause decreased fungal diversity [69]. In addition, disease development can affect the diversity of the phyllosphere fungal community [66]. Contrary to our results, Millberg et al. (2015) found that fungal richness and diversity in the diseased needles of *P*. *sylvestris* significantly increased compared to those of healthy ones [25]. Needles in their study were diseased with multiple pathogens in a broad sense rather than caused by a particular pathogen. Disease symptoms could be of any kind likely to be caused by pathogenic fungi (e.g., spots, bands, discolorations, and dead tips), and they differed from those in our study. We collected needles from diseased trees infected by PWN and showing typical signs of PWD, which could have contributed to the results. Proença et al. (2017) found that the diversity of endophytic wood-colonizing bacteria in the trunk of Portuguese *P. pinaster* trees differed with the severity of the PWD, suggesting the importance of disease development in host-microbial communities [70]. However, samples in our study were collected at one time point in the last stage of PWD, and DNA extraction from tree-trunk samples failed due to technical reasons. Therefore, it is necessary to investigate microbial communities in tree trunks and at different PWD stages for all plant compartments in the future.

Ascomycota and Basidiomycota are the two most abundant fungal taxa in host-microbial communities [71,72,73]. In our study, Basidiomycota was more abundant in the needles of healthy trees, whereas Ascomycota had a higher abundance in diseased trees. The abundance of dominant fungal genera in the needles of healthy trees differed from those in diseased trees, which contributed to the difference in community structures. Interestingly, 17 genera, including *Penicillium,* were enriched in healthy needles, whereas 7 genera, including *Diplodia,* had a higher abundance in the needles of diseased trees. Fungi of the *Penicillium* genus are usually saprophytes, and some species may produce mycotoxins such as citreoviridin [74]. The population of *B*. *xylophilus* was significantly lower than that of the control group on the culture mediums or stem segments of pine trees inoculated with *Penicillium* sp. [51,75]. This also indicated that some products of *Penicillium* sp. may play certain roles in the control of *B*. *xylophilus*, which needs further study. However, some members are also common as endophytes and can improve host resistance to harsh environments. Eight species of *Penicillium* isolated from *P. thunbergii* roots exhibited saline resistance [74]. Notably, *Diplodia* was the dominant genus in the needles of diseased trees. Members of *Diplodia* are among the most globally common and widely distributed pathogens of *Pinus* spp. [14,76], of which *D. sapinea* (Fr.) Fuckel (earlier *D. pinea* and *Sphaeropsis sapinea*) is recognized as an endophyte that can live within host pine branches for years without causing disease. When host health declines, it becomes a weak or latent pine pathogen [77]. Previous studies showed that *D*. *sapinea* is very common in dead pine wood affected by PWN, which can promote the propagation and/or settlement of *B. xylophilus* [51,52]. Therefore, PWD occurrence might be associated with certain host-microbial species, such as *D. sapinea*. Since many other biotic and abiotic factors could affect fungal microhabitat conditions, further investigation is needed to confirm the hypothesis.

No differences in the fungal community were found between healthy and diseased trees in the roots or soil. In our study, we only sampled the main roots and not the fine roots from the trees. Main and fine roots are different in anatomy and function and harbor different microbial communities. More importantly, fine roots can form a symbiosis with certain fungal species, e.g., ECM, which have a close relationship with host health status. Compared to main roots, microbial communities in fine roots might be more sensitive and respond more quickly to a decline in host plant health. PWD can decrease the diversity of root-associated fungi and the colonization of ECM fungi of *P*. *tabulaeformis* [53]. Similarly, a decline in host health can change root exudates, which can directly affect microbial communities in rhizosphere soil rather than bulk soil. A recent study showed that PWN infection can significantly alter the endophytic and rhizospheric microbial community structures of *P*. *massoniana* [54].

Functionally, saprotrophs had a higher abundance in the needles of diseased trees, and symbiotroph abundance was higher in healthy ones, which contributed to the difference in functional structure between the needles of diseased and healthy trees. These indicate that changes in the fungal community in the needles caused by PWD also accordingly shift the functional structure. During the development of PWD, the feeding of nematodes on the resin duct’s epithelial cells of susceptible trees disrupts water conduction, leading to the death of the entire tree [45]. The interruption of nutrient supply can accelerate needle decline. Previous studies showed that the healthy needles of Scots pine harbor a higher diversity of endophytic fungi than diseased ones do, and some can participate in the degradation process of aging needles [78,79]. Senescent spruce needles start to be decomposed while still attached to trees [80]. The initial decomposition stage of needles may be completed by saprophytic fungi that existed as endophytes in living needles [80]. *Hormonema* spp. has a high decomposition ability [80], which might explain the high abundance of the *Hormonema* and *H*. *macroporum* genera in the needles of diseased trees in our study. Moreover, our previous study showed that the abundance of bacteria involved in cellular processes was higher in PWD-affected needles, whereas bacteria with environmental information processing had a higher abundance in healthy needles [55]. The interaction between fungi and bacteria in PWD-affected needles should not be neglected due to their shared niches. A recent study showed that some fungi host their own microbiota on their surfaces and inside the hyphae [5]. Some evidence showed that endophytic bacteria associated with Basidiomycota, Ascomycota, and Mucoromycota can use host lipids, amino acids, and organic acids for their activities [5], suggesting possible nutrient transfer between fungi and its associated bacteria. However, the possible interaction modes between fungi and bacteria in PWD-affected pine need to be further studied to unravel the complex effects of microbes on host plants.

## 5. Conclusions

In conclusion, PWN infection can decrease host fungal diversity in needles. Fungal communities and potential functional structures in the needles of diseased and healthy trees differed, but not in the roots and soil. Basidiomycota and Ascomycota were the dominant phyla in the host fungal community. PWD can increase the abundance of endophytic *Diplodia* in pine needles. Functionally, saprotrophs had a higher abundance in the needles after PWN infection, whereas symbiotroph abundance was higher in healthy ones. The results suggest that PWD can primarily affect fungal communities and potential function in needles. Further investigations on rhizosphere microbes and ectomycorrhizal fungi after PWN infection are needed to understand the effect of PWD on host microbiota.

## Figures and Tables

**Table 1:** Top 10 (> 0.1%) most abundant OTUs showing significant differences in soil (S), roots (R), and needles (N) around and of healthy (H) and diseased (D) trees.

Site	OTUs	Taxonomy	*p*-Value	Abundance Pattern
Soil	OTU00093	*Xenoacremonium*	0.000999	HS < DS
	OTU00127	Trichophaea	0.003996	HS > DS
	OTU00199	Chaetosphaeriaceae	0.028971	HS < DS
	OTU00215	*Helotiales*	0.026973	HS < DS
	OTU00245	*Helotiales*	0.032967	HS > DS
	OTU00222	Bionectriaceae	0.022977	HS > DS
	OTU00230	Hyaloscyphaceae	0.025974	HS > DS
	OTU00219	Chaetosphaeriaceae	0.000999	HS < DS
	OTU00241	Nectriaceae	0.000999	HS > DS
	OTU00261	Thelephoraceae	0.023976	HS < DS
Roots	OTU00154	*Colletotrichum*	0.001998	HR < DR
	OTU00128	*Kuraishia molischiana*	0.030969	HR < DR
	OTU00116	Ascomycota	0.000999	HR > DR
	OTU00171	*Penicillium madriti*	0.036963	HR < DR
	OTU00132	*Cyberlindnera amylophila*	0.004995	HR < DR
	OTU00141	*Boidinia furfuracea*	0.042957	HR > DR
	OTU00178	*Pezicula*	0.037962	HR < DR
	OTU00130	Ascomycota	0.000999	HR > DR
	OTU00168	*Boidinia furfuracea*	0.000999	HR > DR
	OTU00192	Saccharomycetales	0.000999	HR < DR
Needles	OTU00007	*Diplodia*	0.002997	HN < DN
	OTU00011	*Phacidium*	0.002997	HN < DN
	OTU00019	Trichomeriaceae	0.001998	HN > DN
	OTU00025	*Strelitziana*	0.000999	HN > DN
	OTU00026	Trichomeriaceae	0.000999	HN > DN
	OTU00028	*Strelitziana*	0.000999	HN > DN
	OTU00030	*Hormonema_macrosporum*	0.002997	HN < DN
	OTU00040	Trichomeriaceae	0.023976	HN > DN
	OTU00063	Trichomeriaceae	0.002997	HN > DN
	OTU00070	Trichomeriaceae	0.001998	HN > DN

## Data Availability

Raw sequences were deposited at the Sequence Read Archive (SRA) of the National Center for Biotechnology Information (NCBI, https://dataview.ncbi.nlm.nih.gov/object/PRJNA703504) under project accession number PRJNA703504.

## Supplementary Materials

The following are available online at https://www.mdpi.com/article/10.3390/microorganisms9050896/s1, Table S1. The physical and chemical properties in the soil around the healthy and diseased tree. Table S2. PERMANOVA of fungal community structure based on OTU data (a) and pairwise analysis between different samples (b). Table S3. Top 10 most abundant genera in the soil, roots, and needles around and of healthy and diseased trees. Table S4. Top 10 most abundant species in soil, roots, and needles around and of healthy and diseased trees. Table S5. PERMANOVA of fungal functional structure based on FUNGUILD data (a) and pairwise analysis between different samples (b). Figure S1. Rarefaction curve showing observed OTUs and sequence depth in (a) needles, (b) roots, and (c) soil around and of healthy and diseased trees.
