# Supplementary material for: The Impact of Pine Wood Nematode Infection on the Host Fungal Community
Source: Microorganisms. 2021 Apr 22;9(5):896. doi: 10.3390/microorganisms9050896 (PMC8146488; doi:10.3390/microorganisms9050896)
Supplement: Supplementary file 1 [file microorganisms-09-00896-s001.zip › microorganisms-1165692-supplementary.pdf]

Supplementary Table

Table S1*. The physical and chemical properties in soil around the healthy and diseased tree

Samples	Soil Water Content	Soil Organic Matter (g/kg)	pH	Microbial Biomass Carbon	Total Nitrogen (g/kg)
Soil around Diseased tree	0.32 ± 0.04	149.72 ± 6.70	5.57 ± 0.08 *	232.45 ± 5.94 *	21.74 ± 0.62 *
Soil around Healthy tree	0.30 ± 0.01	141.49 ± 8.24	5.16 ± 0.01	170.38 ± 0.94	19.28 ± 0.53

The values are shown as means ± standard deviation (n = 27). * $P < 0.05$, significant difference in the global Kruskal–Wallis test.
*****Table was cited from Ma et al. (2020).

Table S2. PERMANOVA analysis of fungal community structure based on the OTUs data (a) and pairwise analysis between different samples (b)

(a)

**Source(sources of variation**	**Df (degrees of freedom)**	**SS (sums of squars)**	**MS (mean squars)**	**Pseudo-F**	**P (perm)**	**Unique perms**
Plant compartment	2	85362	42681	19,234	0,001	997
Disease	1	3825,5	3825,5	1,7239	0,063	997
Plant compartment x Disease	2	7555,2	3777,6	1,7024	0,03	999
Res (residual)	48	1,0651E+05	2219			
Total	53	2,0326E+05				

Factors: Plant compartment (3 levels) and Disease (2 levels)
Permutation method: Unrestricted permutation of raw data
Number of permutations: 999

(b)

**Groups**	**T**	**P (perm)**	**Unique perms**
Root in diseased tree, Root in healthy tree	1.006	0.416	968
Root in diseased tree, Needle in diseased tree	3.108	0.001	981
Root in diseased tree, Needle in healthy tree	3.216	0.001	980
Root in diseased tree, Soil around diseased tree	2.645	0.001	969
Root in diseased tree, Soil around healthy tree	2.508	0.001	977
Root in healthy tree, Needle in diseased tree	3.420	0.001	981
Root in healthy tree, Needle in healthy tree	3.531	0.001	969
Root in healthy tree, Soil around diseased tree	2.642	0.001	979
Root in healthy tree, Soil around healthy tree	2.476	0.001	980
Needle in diseased tree, Needle in healthy tree	2.244	0.001	984
Needle in diseased tree, Soil around diseased tree	3.993	0.001	978
Needle in diseased tree, Soil around healthy tree	3.805	0.001	972
Needle in healthy tree, Soil around diseased tree	4.134	0.001	985
Needle in healthy tree, Soil around healthy tree	3.951	0.001	977
Soil around diseased tree, Soil around healthy tree	0.770	0.992	978

Permutation method: Unrestricted permutation
Number of permutations: 999

Table S3. The top 10 most abundant genera in the soil, roots and needles around/of the healthy and diseased trees

Phylum	Total (%)	Soil (%)		Roots (%)		Needles (%)	
		**Diseased**	**Healthy**	**Diseased**	**Healthy**	**Diseased**	**Healthy**
Mortierella	9.3±11.99	14.4±11.13	16.57±12.92	0.04±0.03	0.08±0.05		
Delicatula	3.94±9.43			13.12±11.35	26.21±8.31		0.02±0.02
Trichoderma	3.06±4.51	6.70±5.68	3.40±3.34	0.10±0.08	0.18±0.18		
Diplodia	1.93±7.80	0.01±0.02				19.14±17.54	0.11±0.09**
Solicoccozyma	1.89±2.55	3.43±2.79	2.85±2.49		0.01±0.01		
Cenangium	1.85±7.11					17.98±15.33	0.54±1.32
Strelitziana	1.72±5.56	0.05±0.14	0.01±0.02			1.17±1.3	15.89±9.45**
Penicillium	1.66±2.10	2.90±2.53	2.42±1.83	0.05±0.05	0.22±0.13**	0.01±0.01	0.35±0.32**
Phacidium	1.61±5.90	0.04±0.05	0.05±0.06			15.76±11.73	0.08±0.08**
Sebacina	1.04±2.13	0.10±0.09	3.22±2.88	0.01±0.01	0.40±0.30		

The values were shown as mean ± standard deviation (n=9, except for soil n=27). * *P* < 0.05, ** *P* < 0.01, significant difference in LSD (least-significant difference) test. The blank in the Tables means that the relative abundance of that genera is not exist in the samples.

Table S4. The top 10 most abundant species in the soil, roots and needles around/of the healthy and diseased trees

Phylum	Total (%)	Soil (%)		Roots (%)		Needles (%)	
		**Diseased**	**Healthy**	**Diseased**	**Healthy**	**Diseased**	**Healthy**
Mortierella_humilis	13.91±17.03	20.36±16.26	24.87±17.88	0.38±0.15	3.02±2.73		
Mortierella_minutissima	5.54±6.88	7.82±7.04	10.19±6.81		1.39±1.37		
Pestalotiopsis_rhododendri	3.97±9.38	1.68±1.65	0.55±0.56**	0.02±0.02	0.04±0.31**	29.42±12.02	3.20±2.34**
Solicoccozyma_terrea	3.93±5.15	6.66±5.46	6.39±5.11		0.15±0.21		
Hormonema_macrosporum	2.14±5.33					16.04±5.44	5.35±4.1
Humicola_olivacea	1.53±2.32	3.38±3.15	1.36±1.47	0.02±0.04	1.08±1.00		
Trichoderma_atroviride	1.38±2.1	3.20±2.94	1.07±0.96	0.38±0.55	0.63±0.55		
Solicoccozyma_terricola	1.31±2.16	2.94±3.18	1.30±0.94		0.36±0.47		
Sclerostagonospora_lathyri	0.81±2.39					1.78±1.75	6.28±4.47
Boidinia_furfuracea	0.76±2.32			0.16±0.17	7.46±1.91		

The values were shown as mean ± standard deviation (n=9, except for soil n=27). * *P* < 0.05, ** *P* < 0.01, significant difference in LSD (least-significant difference) test. The blank in the Tables means that the relative abundance of that species is not exist in the samples.

Table S5. PERMANOVA analysis of fungal functional structure based on the FUNGuild data (a) and pairwise analysis between different samples (b)

(a)

**Source(sources of variation**	**Df (degrees of freedom)**	**SS (sums of squars)**	**MS (mean squars)**	**Pseudo-F**	**P (perm)**	**Unique perms**
Plant compartment	2	90281	45141	22,539	0,001	997
Disease	1	3992,4	3992,4	1,9934	0,04	998
Plant compartment x Disease	2	7719,1	3859,5	1,9271	0,021	998
Res (residual)	48	96133	2002,8			
Total	53	1,9813E+05				

Factors: Plant compartment (3 levels) and Disease (2 levels)
Permutation method: Unrestricted permutation of raw data
Number of permutations: 999

(b)

**Groups**	**T**	**P (perm)**	**Unique perms**
Needle in diseased tree, Needle in healthy tree	2.2441	0.001	971
Needle in diseased tree, Root in diseased tree	3.1076	0.001	978
Needle in diseased tree, Root in healthy tree	3.4202	0.001	971
Needle in diseased tree, Soil around diseased tree	3.9934	0.001	974
Needle in diseased tree, Soil around healthy tree	3.8048	0.001	972
Needle in healthy tree, Root in diseased tree	3.2158	0.001	978
Needle in healthy tree, Root in healthy tree	3.5313	0.001	986
Needle in healthy tree, Soil around diseased tree	4.134	0.001	979
Needle in healthy tree, Soil around healthy tree	3.9508	0.001	988
Root in diseased tree, Root in healthy tree	1.006	0.406	975
Root in diseased tree, Soil around diseased tree	2.6452	0.001	973
Root in diseased tree, Soil around healthy tree	2.5076	0.001	974
Root in healthy tree, Soil around diseased tree	2.6423	0.001	983
Root in healthy tree, Soil around healthy tree	2.4764	0.002	979
Soil around diseased tree, Soil around healthy tree	0.77017	0.99	976

Permutation method: Unrestricted permutation
Number of permutations: 999

Supplementary Figure

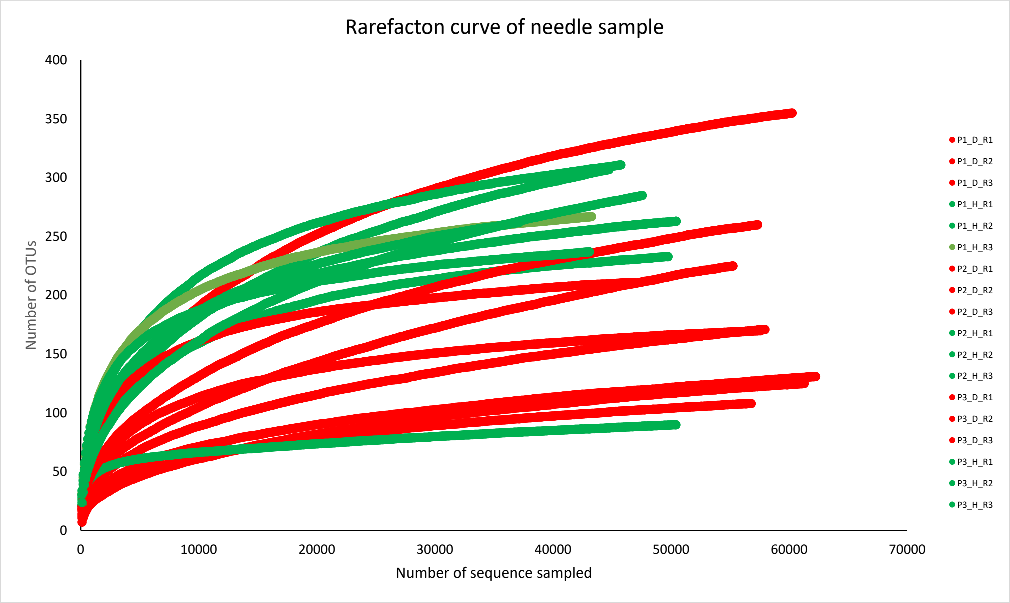

(a)

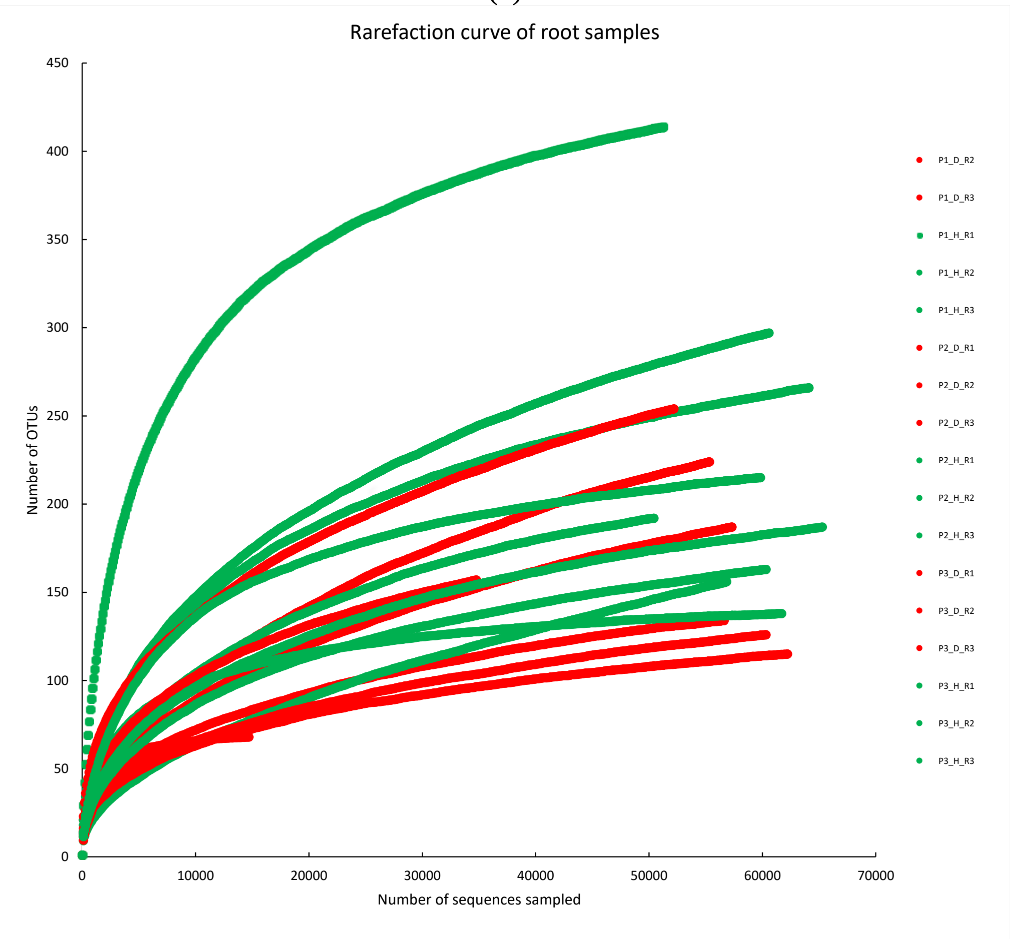

(b)

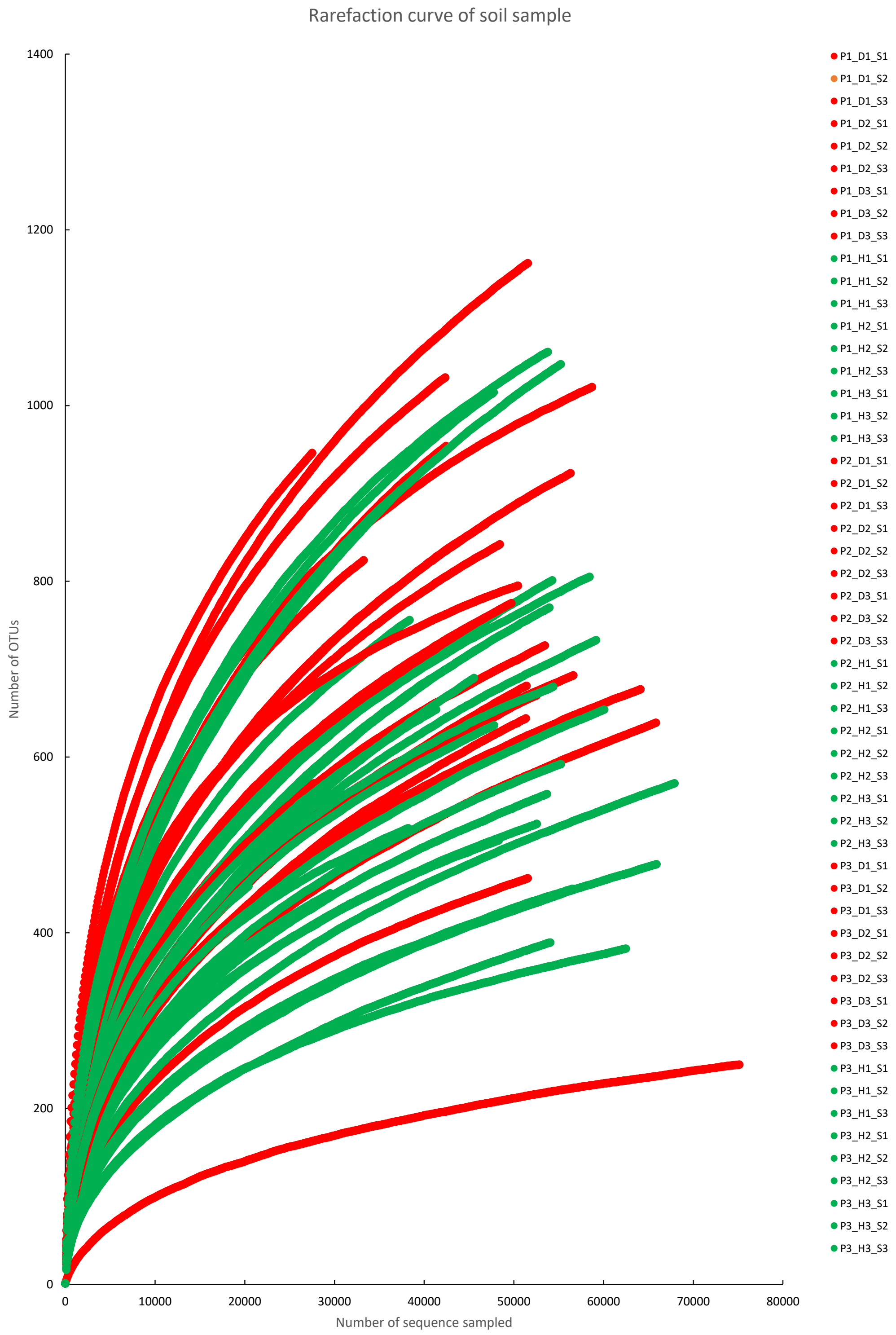

(c)

Figure S1. Rarefaction curve showing the observed OTUs and sequence depth in the needles (a), roots (b) and soil (c) of and around the healthy and diseased trees. Abbreviations in the legend: P: Plot, D: Diseased tree; H: Healthy tree; R: Root; N: Needle; S: Soil. The number after the abbreviation showing the three replicates (1, 2 and 3). Red and green color indicating diseased and healthy trees, respectively.
